# Pancreatic kallikrein protects against diabetic retinopathy in KK Cg-*A*^*y*^/J and high-fat diet/streptozotocin-induced mouse models of type 2 diabetes

**DOI:** 10.1007/s00125-019-4838-9

**Published:** 2019-03-05

**Authors:** Ying Cheng, Xiaochen Yu, Jie Zhang, Yunpeng Chang, Mei Xue, Xiaoyu Li, Yunhong Lu, Ting Li, Ziyu Meng, Long Su, Bei Sun, Liming Chen

**Affiliations:** 1 NHC Key Laboratory of Hormones and Development, Tianjin Key Laboratory of Metabolic Diseases, Metabolic Diseases Hospital & Institute of Endocrinology, Tianjin Medical University, Tianjin, 300070 China; 2 The Second Hospital of Tianjin Medical University, Tianjin, China

**Keywords:** Apoptosis, Diabetic retinopathy, Inflammation, Oxidative stress, Pancreatic kallikrein

## Abstract

**Aims/hypothesis:**

Many studies have shown that tissue kallikrein has effects on diabetic vascular complications such as nephropathy, cardiomyopathy and neuropathy, but its effects on diabetic retinopathy are not fully understood. Here, we investigated the retinoprotective role of exogenous pancreatic kallikrein and studied potential mechanisms of action.

**Methods:**

We used KK Cg-*A*^*y*^/J (KKAy) mice (a mouse model of spontaneous type 2 diabetes) and mice with high-fat diet/streptozotocin (STZ)-induced type 2 diabetes as our models. After the onset of diabetes, both types of mice were injected intraperitoneally with either pancreatic kallikrein (KKAy + pancreatic kallikrein and STZ + pancreatic kallikrein groups) or saline (KKAy + saline and STZ + saline groups) for 12 weeks. C57BL/6J mice were used as non-diabetic controls for both models. We analysed pathological changes in the retina; evaluated the effects of pancreatic kallikrein on retinal oxidative stress, inflammation and apoptosis; and measured the levels of bradykinin and B1 and B2 receptors in both models.

**Results:**

In both models, pancreatic kallikrein improved pathological structural features of the retina, increasing the thickness of retinal layers, and attenuated retinal acellular capillary formation and vascular leakage (*p* < 0.05). Furthermore, pancreatic kallikrein ameliorated retinal oxidative stress, inflammation and apoptosis in both models (*p* < 0.05). We also found that the levels of bradykinin and B1 and B2 receptors were increased after pancreatic kallikrein in both models (*p* < 0.05).

**Conclusions/interpretation:**

Pancreatic kallikrein can protect against diabetic retinopathy by activating B1 and B2 receptors and inhibiting oxidative stress, inflammation and apoptosis. Thus, pancreatic kallikrein may represent a new therapeutic agent for diabetic retinopathy.

**Electronic supplementary material:**

The online version of this article (10.1007/s00125-019-4838-9) contains peer reviewed but unedited supplementary material, which is available to authorised users.

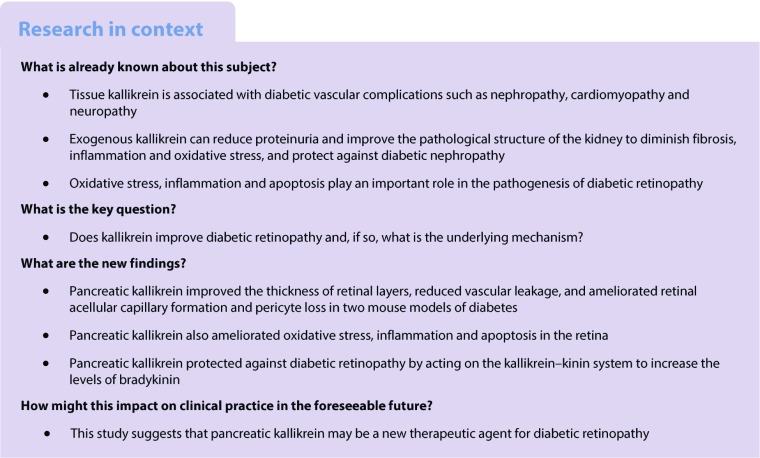



## Introduction

Diabetic retinopathy is one of the most common microvascular complications of diabetes and an important cause of non-traumatic blindness in adults [1, 2]. Diabetic retinopathy develops in two stages: non-proliferative and proliferative. The early stages mainly involve vascular cell loss, vascular leakage and the destruction of the blood–retinal barrier. As the disease progresses, neovascularisation gradually occurs and the proliferative diabetic retinopathy stage begins. Among the types of diabetic retinopathy, diabetic macular oedema is the most common and is the main cause of visual impairment and blindness in people with diabetes. The main pathophysiology involves increased vascular leakage and accumulation of fluid in the macula, leading to macular oedema and increased retinal thickness [3, 4]. Many studies have shown that oxidative stress, inflammation and apoptosis play important roles in the early stages of diabetic retinopathy [5–8]. Therefore, inhibiting oxidative stress, inflammation and apoptosis may prevent diabetic retinopathy.

Kallikrein is a serine protease that exists as serum kallikrein and tissue kallikrein, which are key enzymes of the kallikrein–kinin system (KKS) [9]. Many studies have shown that the occurrence of diabetic microvascular complications is associated with the KKS, especially kallikrein. Montanari et al found that kallikrein promotes glucose utilisation and lipid metabolism and improves diabetic cardiomyopathy [10]. Some studies have reported that exogenous kallikrein reduces proteinuria and improves the pathological structure of the kidney, fibrosis, inflammation and oxidative stress to protect against diabetic nephropathy [11, 12]. However, the effects of plasma and tissue kallikrein on diabetic retinopathy are controversial. Clermont et al demonstrated that plasma kallikrein contributes to retinal vascular permeability [13], while other studies have shown that tissue kallikrein improves diabetic retinopathy by inhibiting retinal vascular permeability and vascular endothelial growth factor (VEGF) increases in rat models of diabetes [14].

In this study, we evaluated the retinal protective role of pancreatic kallikrein in KKAy mice (a mouse model of spontaneous type 2 diabetes) and a mouse model of high-fat diet (HFD)/streptozotocin (STZ)-induced type 2 diabetes. We investigated whether pancreatic kallikrein could alleviate diabetic retinopathy by changing the KKS and improving pathological structural features of the retina, and explored the potential mechanisms of action.

## Methods

### KK Cg-*A*^*y*^/J mice

Male KK Cg-*A*^*y*^/J (KKAy) mice were originally developed by Japanese scholars, who transferred the *Ay* gene into KK mice [15]. After they had acclimated for 2 weeks, the mice were randomly divided into two groups. One group underwent daily i.p. injections of saline (154 mmol/l NaCl; *n* = 16, 10 ml/kg, KKAy + saline group), while the other group underwent daily i.p. injections of pancreatic kallikrein (Qianhong Biochemical Pharmaceutical Company, Changzhou, China) (*n* = 16, 40 U/kg, KKAy + pancreatic kallikrein group) for 12 weeks. Both groups of mice were fed KK feed. In addition, age-matched C57BL/6J mice (*n* = 16, C57 group) were used as controls. See electronic supplementary materials (ESM) Methods for details.

### HFD/STZ-induced type 2 diabetic mice

For the second mouse model of type 2 diabetes, male C57BL/6J mice were chosen and diabetes was induced as previously described [16]. First, 16 mice were randomly chosen to receive a standard diet (normal control group), while the others were fed an HFD (*n* = 35) for 12 weeks. Subsequently, mice in the HFD group were injected with STZ (30 mg/kg; Sigma-Aldrich, St Louis, MO, USA) i.p. for 7 consecutive days, while the mice in the normal control group were injected i.p. with citrate–phosphate buffer. One week after the final injection, blood glucose levels were tested and mice with random blood glucose levels above 16.7 mmol/l were considered to be type 2 diabetic mice. The diabetic mice were then randomly separated into two groups, one of which was treated with pancreatic kallikrein (*n* = 16, STZ + pancreatic kallikrein group) and the other with saline (*n* = 16, STZ + saline group). The injection method, dose and time of pancreatic kallikrein administration were the same as for the KKAy mice. Three mice were excluded because their blood glucose did not reach the threshold. Body weight and blood glucose were measured weekly until the end of the study. After 12 weeks of pancreatic kallikrein treatment, all mice were killed. See ESM Methods for details.

All mice were housed two per cage in polycarbonate cages with corncob bedding and maintained on a 12 h light/12 h dark cycle and 10% humidity at 20 ± 4°C with ad libitum access to diet. All animal studies were conducted in accordance with the Guide for the Care and Use of Laboratory Animals of the National Institutes of Health, as well as the Animal Welfare Act guidelines. The protocols were approved by the ethical committee of Tianjin Medical University.

### IPGTT

In the HFD/STZ-induced diabetic mice, an IPGTT was performed after 12 weeks of an HFD to assess insulin resistance. See ESM Methods for details.

### Optical coherence tomography

After 12 weeks of treatment, all mice were subjected to optical coherence tomography (OCT). Mice were weighed and anaesthetised using an i.p. injection of 1% (vol./vol.) sodium pentobarbital (30 mg/kg), and 0.5% tropicamide solution was used for eye dilation. The mice were placed on an adjustable box for spectral domain OCT (Heidelberg Engineering, Heidelberg, Germany). OCT scanning was initially directed from the posterior pole to the central portion of the mouse eye, which was facilitated by aiming the laser beam of the scanner. Retinal thickness was evaluated using Image-Pro Plus 6.0 analysis software (Media Cybernetics, Rockville, MD, USA).

### H&E staining

Eyeballs were fixed in 4% paraformaldehyde, embedded in paraffin and cut into 4 μm sections. As it is thought that retinal layer thickness varies throughout the retina, all sections were cut in the same orientation and always through the optic nerve head, and the regions were quantified at a constant distance from the optic nerve head. After deparaffinisation, H&E staining was performed and observed under a light microscope. Retinal thickness was evaluated using Image-Pro Plus 6.0 analysis software.

### Immunohistochemistry

Paraffin-embedded retinas were cut into 4 μm sections for immunohistochemistry analysis (see ESM Methods for details). The stained sections were observed and imaged under a light microscope, and staining was quantified using Image-Pro Plus 6.0 analysis software.

### Retinal trypsin digestion

Retinal trypsin digestion was performed as previously described [17]. Acellular vessels and pericytes were quantified from six random fields per retina under ordinary light microscopy according to a documented protocol [18]. See ESM Methods for details.

### Retinal vascular immunofluorescence staining

Next, we performed retinal immunofluorescence staining and observed the results under a laser scanning confocal microscope (LSM 710, Carl Zeiss, Oberkochen, Germany). Retinal capillary density was analysed using Image J software (National Institutes of Health, Bethesda, MD, USA). See ESM Methods for details.

### Reactive oxygen species (ROS) detection

Cryosections (4 μm) of eyecups were stained with dihydroethidium (Vigorous Biotechnology, Beijing, China) for 45 min at 37°C as previously described [19]. Fluorescence images were observed under a fluorescence microscope and positive staining was quantified using Image-Pro Plus 6.0 analysis software.

### TUNEL assay

Apoptosis detection was performed using a TUNEL apoptosis detection kit (biotin-labelled POD method; Universal, Nanjing, China) according to the manufacturer’s instructions. See ESM Methods for details.

### ELISA

Serum bradykinin levels were determined by using an ELISA kit (JL12234, Jianglaibio, Shanghai, China). All ELISA steps were conducted in accordance with the manufacturer’s protocols.

### Western blot analysis

Western blotting was performed to determine the retinal expression of NADPH oxidase 2 (NOX2), superoxide dismutase 2 (SOD2), IL-1β, TNF-α, VEGF, cleaved caspase 3, BAX, Bcl-2, B1 receptor (B1R) and B2 receptor (B2R). Densitometry analyses of the bands were performed using Image J software. See ESM Methods for details.

### Quantitative real-time PCR (qPCR)

qPCR was used to determine the relative expression levels of mRNA (see ESM Methods for details). Primer sequences for *B1r* (also known as *Bdkrb1*), *B2r* (also known as *Bdkrb2*) and *Klk1* (which encodes kallikrein 1) are shown in ESM Table 1.

### Statistical analysis

Statistical analysis was performed using Prism 5.0 software (GraphPad, La Jolla, CA, USA). Values are shown as the means ± SEM. The powers of each experiment were calculated using PASS software (PASS 11, Kaysville, UT, USA) and are shown in ESM Table 2. The significance of differences among different groups was evaluated using ANOVA; *p <* 0.05 was considered statistically significant.

## Results

### Animal characteristics

Data on body weight and blood glucose, creatinine, alanine aminotransferase (ALT) and triacylglycerol levels after 12 weeks of pancreatic kallikrein treatment for the KKAy mice are summarised in ESM Table 3. Compared with those of the C57 group, the body weight and blood glucose, creatinine, ALT and triacylglycerol levels of the KKAy + saline group were significantly elevated (*p* < 0.05). However, these variables did not change significantly with pancreatic kallikrein treatment. The characteristics of the HFD/STZ-induced type 2 diabetic mice are presented in ESM Table 4. Unlike for the KKAy mice, the ALT level in the STZ + pancreatic kallikrein group was decreased compared with the STZ + saline group, although there was no statistical difference (*p* = 0.18); there were also no significant differences in creatinine levels among the STZ + pancreatic kallikrein, STZ + saline and normal control groups (*p* = 0.0839).

### Pancreatic kallikrein protects against retinal structure changes

**Fig. 1:**
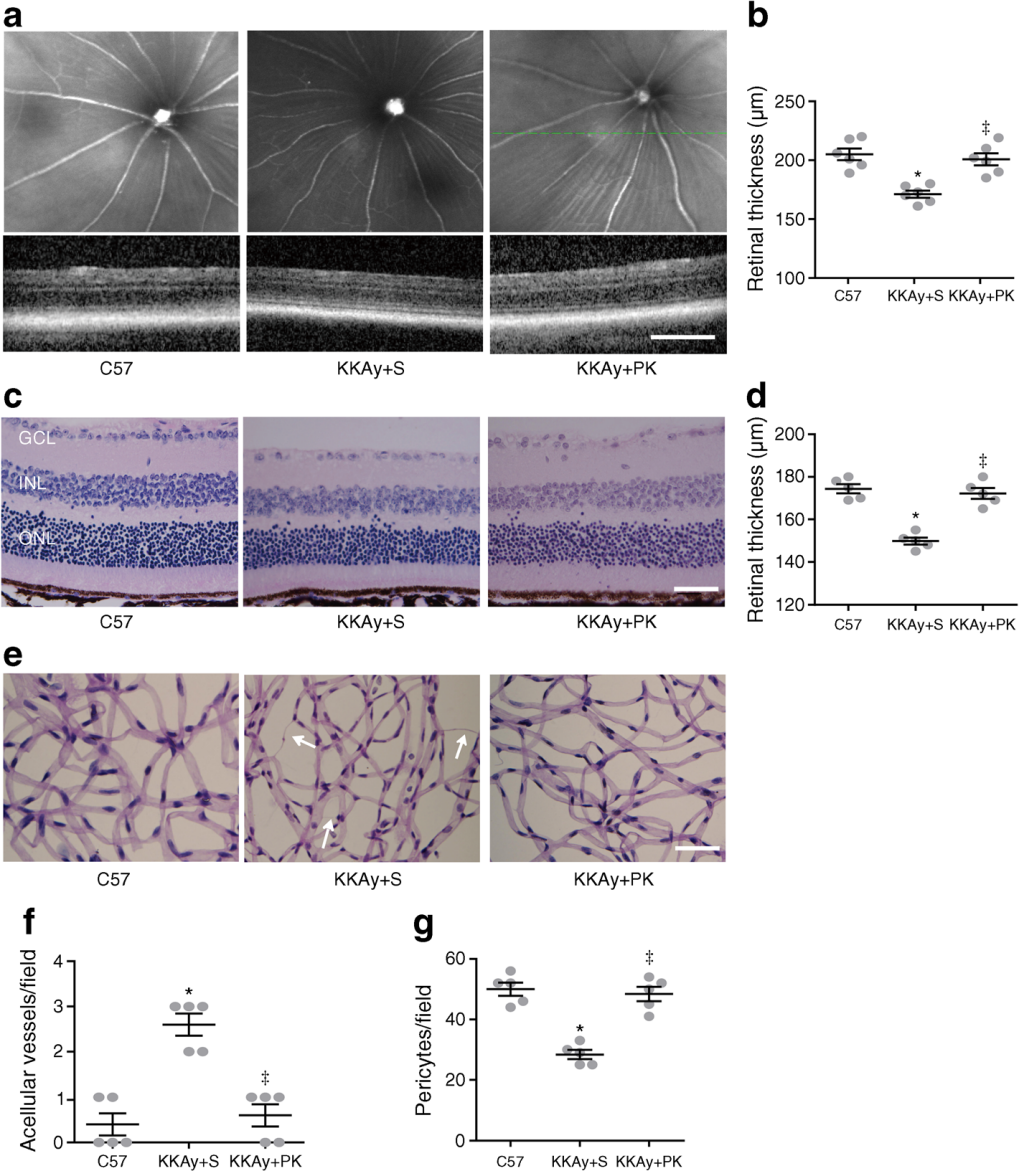
Pancreatic kallikrein protects the retinal structure in KKAy mice. (**a**) Representative photomicrographs from retinal OCT in C57 and KKAy mice. The green line indicates the position near the optic nerve head. Scale bar, 200 μm; *n*=6 experimental samples per group. (**b**) Quantification of the total retinal thickness in the circular area close to the optic nerve head. (**c**) Representative images of retinal H&E staining in C57 and KKAy mice and (**d**) quantification of the total retinal thickness in the circular area around the optic nerve head. Scale bar, 50 μm. (**e**) Representative images of retinal trypsin digestion assay in C57 and KKAy mice. White arrows indicate acellular vessels. Scale bar, 50 μm. (**f**, **g**) Acellular vessels and pericytes were quantified in flat-mounted retinas of C57 and KKAy mice. Data are expressed as means ± SEM; *n*=5 experimental samples per group. *p<0.05 vs the C57 group; ^‡^*p*<0.05 vs the KKAy + saline (S) group. GCL, ganglion cell layer; INL, inner nuclear layer; ONL, outer nuclear layer; PK, pancreatic kallikrein; S, saline

**Fig. 2:**
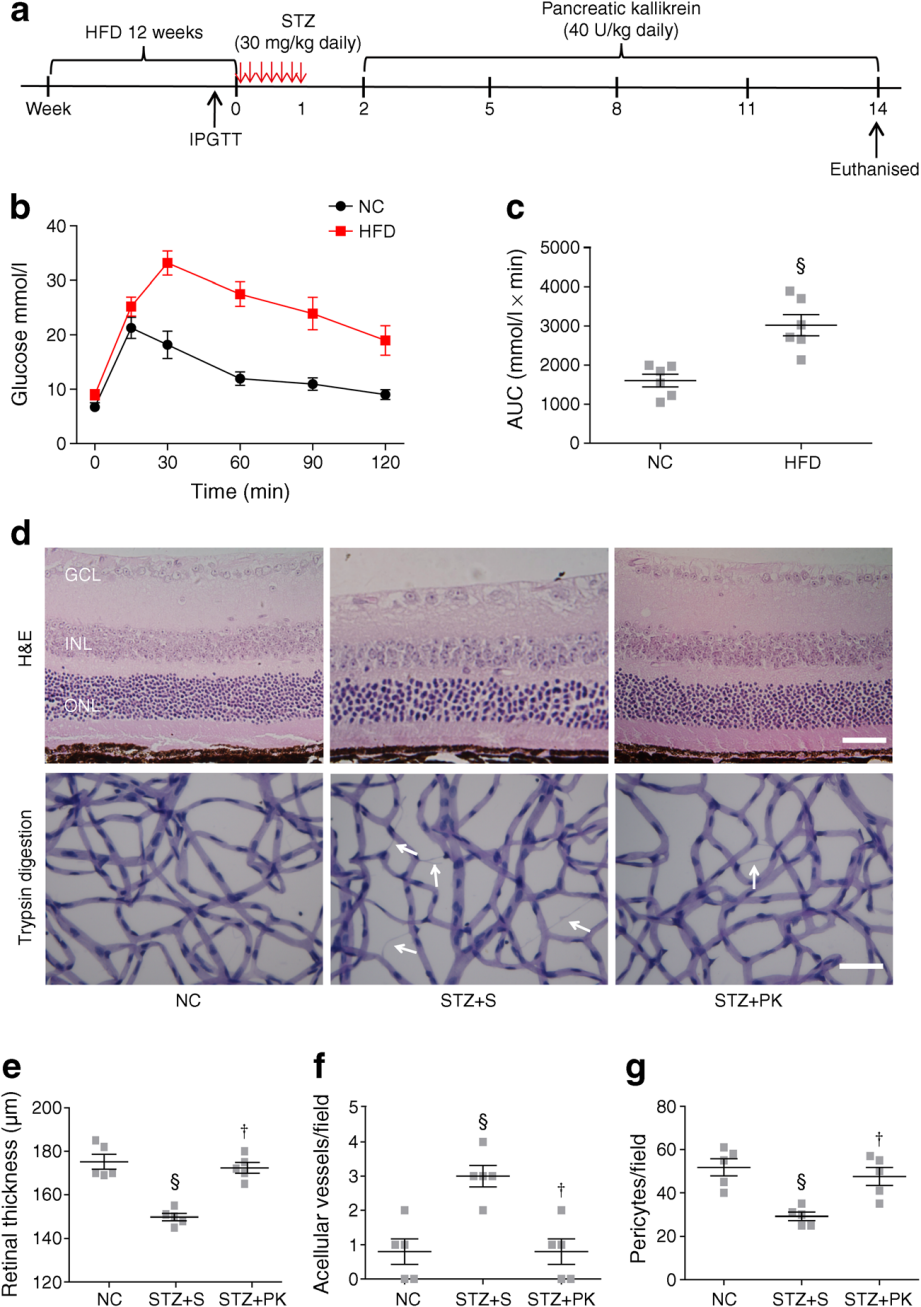
Pancreatic kallikrein protects the retinal structure in HFD/STZ-induced type 2 diabetic mice. (**a**) Experimental procedure for HFD/STZ-induced type 2 diabetes. (**b**, **c**) Curve and AUC for the results of the IPGTT; *n*=6 experimental samples per group. (**d**) Representative images of retinal H&E staining and a trypsin digestion assay in HFD/STZ-induced type 2 diabetic mice. The white arrows indicate acellular vessels. Scale bars, 50 μm. (**e**) Quantification of the total retinal thickness in the circular area around the optic nerve head. (**f**, **g**) Acellular vessels and pericytes were quantified in flat-mounted retinas. Data are expressed as means ± SEM; *n*=5 experimental samples per group. ^§^*p*<0.05 vs the normal control (NC) group; ^†^*p*<0.05 vs the STZ + saline (S) group. GCL, ganglion cell layer; INL, inner nuclear layer; NC, normal control; ONL, outer nuclear layer; PK, pancreatic kallikrein; S, saline

Previous studies have demonstrated that retinal layer thickness is significantly decreased in diabetic mice [20]. To verify whether pancreatic kallikrein has an effect on the structure of the diabetic retina, we first performed OCT, a non-invasive test to observe the retinal thickness in live mice. The results showed that the retinal layers were significantly thinned in the KKAy + saline group and that pancreatic kallikrein reversed this change. This finding was confirmed by semiquantitative analysis (Fig. 1a, b). Next, we performed H&E staining after tissue harvest. All samples were cut at a constant distance from the optic nerve head to increase comparability. Photomicrographs of H&E staining revealed that the retinal layers were significantly thinner in the KKAy + saline group than in the C57 group, but that this effect was reversed by pancreatic kallikrein (Fig. 1c, d). Fig. 2a shows the entire experimental procedure for the HFD/STZ-induced type 2 diabetic mice. In this model, the IPGTT showed that after 12 weeks of HFD feeding, the HFD group had obvious insulin resistance compared with the normal control group (*p* < 0.05) (Fig. 2b, c). In HFD/STZ-induced type 2 diabetic mice, H&E staining also indicated that pancreatic kallikrein could reverse the observed decrease in retinal thickness (Fig. 2d, e). This finding was consistent with the results for KKAy mice.

### Pancreatic kallikrein attenuates vascular damage in the retina

A retinal trypsin digestion assay was performed to investigate acellular capillary formation and the number of pericytes. Compared with the C57 group, the KKAy + saline group had significant increases in retinal acellular capillaries and fewer pericytes. However, pancreatic kallikrein reduced these pathological changes (Fig. 1e). This finding was confirmed by semiquantitative analysis (Fig. 1f, g). Similarly, in the STZ + saline group, we observed acellular capillaries and a loss of pericytes, while pancreatic kallikrein reversed these pathological changes (Fig. 2d, f, g).

**Fig. 3:**
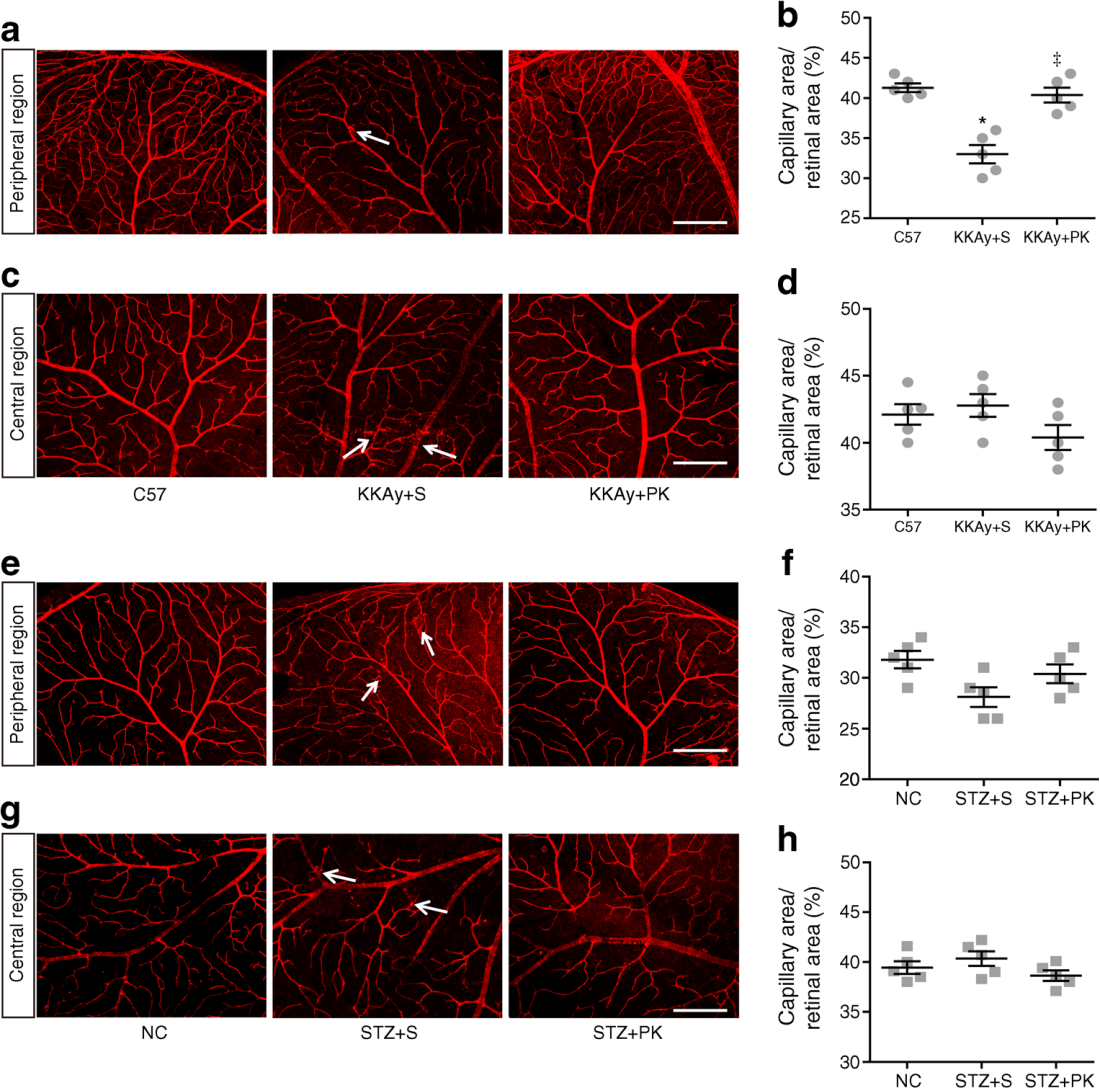
Effect of pancreatic kallikrein on retinal vessels in KKAy and HFD/STZ-induced type 2 diabetic mice. (**a**) Representative immunofluorescence images of peripheral area vessels of the retina and (**b**) quantification of peripheral area vessel densities; (**c**) representative immunofluorescence images of central area vessels of the retina and (**d**) quantification of central area vessel densities in the C57, KKAy + saline (S) and KKAy + pancreatic kallikrein (PK) groups. (**e**) Representative immunofluorescence images of peripheral area vessels of the retina and (**f**) quantification of peripheral area vessel densities; (**g**) representative immunofluorescence images of central area vessels of the retina and (**h**) quantification of central area vessel densities in the normal control, STZ + S and STZ + PK groups. White arrows indicate vascular leakage. Scale bars, 200 μm. Data are expressed as means ± SEM; *n*=5 experimental samples per group. *p<0.05 vs the C57 group; ^‡^*p*<0.05 vs the KKAy + S group; NC, normal control; PK, pancreatic kallikrein; S, saline

Next, we used laser confocal microscopy to analyse the retinal capillary density. We found that capillary density in the peripheral region of the retina was significantly lower in the KKAy + saline group than in the C57 group, and that pancreatic kallikrein attenuated this reduction in capillary density (Fig. 3a, b). In contrast, there was no significant change in the density of capillaries in the central region of the retina (Fig. 3d), but vascular leakage was observed in the KKAy + saline group, and pancreatic kallikrein improved the leakage (Fig. 3c). Similarly, we analysed the retinal capillary density in HFD/STZ-induced type 2 diabetic mice. There were no significant changes in the density of retinal capillaries in these three groups in either the peripheral or central regions, but vascular leakage was observed in the STZ + saline group in both the peripheral and central regions, and pancreatic kallikrein improved this phenomenon (Fig. 3e–h).

### Pancreatic kallikrein ameliorates retinal oxidative stress

**Fig. 4:**
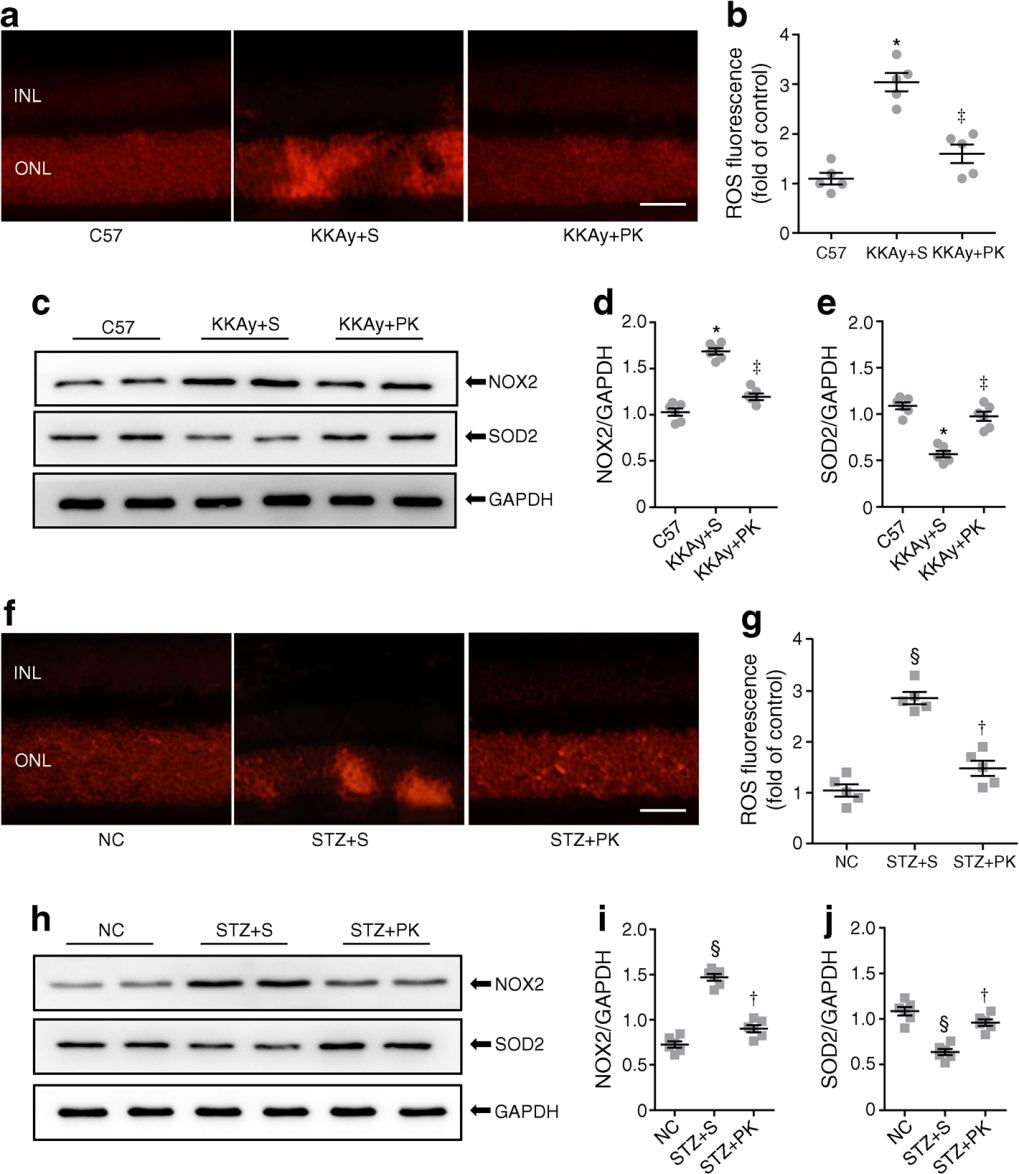
Pancreatic kallikrein suppresses retinal oxidative stress in KKAy and HFD/STZ-induced type 2 diabetic mice. (**a**, **f**) The production of ROS, measured by dihydroethidium staining and (**b**, **g**) quantification of the fluorescence intensity in each experimental group shown. Scale bars, 50 μm. *n*=5 experimental samples per group. (**c**, **h**) Western blot analysis of NOX2 and SOD2 protein and (**d**, **e**, **i**, **j**) densitometric analysis for band intensities normalised to GAPDH in each experimental group shown. Data are expressed as fold of the control for that experiment (C57 or normal control [NC]), or as a ratio, and as means ± SEM; *n*=6 experimental samples per group.*p<0.05 vs the C57 group; ^‡^*p*<0.05 vs the KKAy + saline (S) group; ^§^*p*<0.05 vs the NC group; ^†^*p*<0.05 vs the STZ + S group. GAPDH, glyceraldehyde 3-phosphate dehydrogenase; INL, inner nuclear layer; NC, normal control; ONL, outer nuclear layer; PK, pancreatic kallikrein; S, saline

The above results indicated that pancreatic kallikrein could improve pathological structural changes in the retina. Since oxidative stress is the main pathological process in diabetic retinopathy [5], we wanted to verify whether pancreatic kallikrein has any effect on retinal oxidative stress. First, we used dihydroethidium staining to detect the production of ROS. The results showed that ROS expression was markedly higher in the KKAy + saline group than in the C57 group, while pancreatic kallikrein markedly reduced ROS levels nearly back to normal levels (Fig. 4a). This result was confirmed by semiquantitative analysis (Fig. 4b). Next, we examined other biomarkers of oxidative stress. We found that NOX2 expression was higher in the KKAy + saline group than in the C57 group. However, NOX2 expression was decreased in the KKAy+ pancreatic kallikrein group (Fig. 4c, d). SOD2 is an important indicator of antioxidant activity. Our results showed that the expression of SOD2 in the KKAy + saline group was significantly decreased, while pancreatic kallikrein reversed this change (Fig. 4c, e). The results regarding the levels of ROS, NOX2 and SOD2 in HFD/STZ-induced diabetic mice were consistent with those in KKAy mice (Fig. 4f–j).

### Pancreatic kallikrein suppresses retinal inflammation

**Fig. 5:**
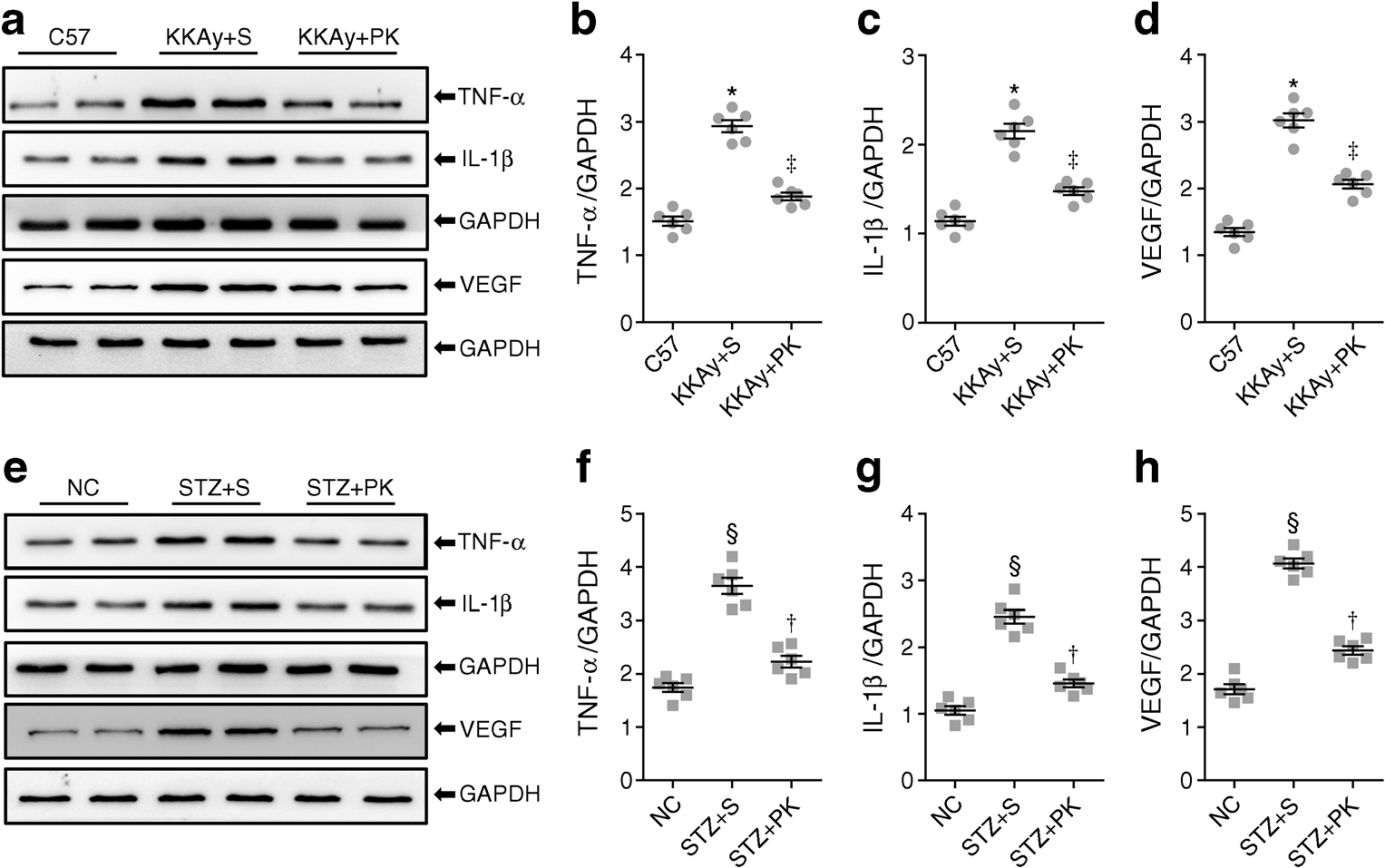
Pancreatic kallikrein ameliorates retinal inflammation in KKAy and HFD/STZ-induced type 2 diabetic mice. (**a**, **e**) Western blot analysis of TNF-α, IL-1β and VEGF protein, with GAPDH run for each gel, and (**b**–**d**, **f**–**h**) densitometric analysis for band intensities normalised to GAPDH in each experimental group. Data are expressed as means ± SEM; *n*=6 experimental samples per group. *p<0.05 vs the C57 group; ^‡^*p*<0.05 vs the KKAy + saline (S) group; ^§^*p*<0.05 vs the normal control (NC) group; ^†^*p*<0.05 vs the STZ + S group. GAPDH, glyceraldehyde 3-phosphate dehydrogenase; NC, normal control; PK, pancreatic kallikrein; S, saline

Chronic inflammation is a major pathogenic factor in diabetic retinopathy [6]. To verify whether pancreatic kallikrein improved retinal inflammation, we examined the inflammatory factors TNF-α, IL-1β and VEGF. Compared with C57 mice, KKAy + saline mice showed significantly increased TNF-α, IL-1β and VEGF levels, which were reduced in KKAy + pancreatic kallikrein mice (Fig. 5a–d). Similar western blot results for TNF-α, IL-1β and VEGF in HFD/STZ-induced diabetic mice are shown in Fig. 5e–h. Other inflammation indicators are shown in ESM Fig. 1.

### Pancreatic kallikrein improves retinal apoptosis

**Fig. 6:**
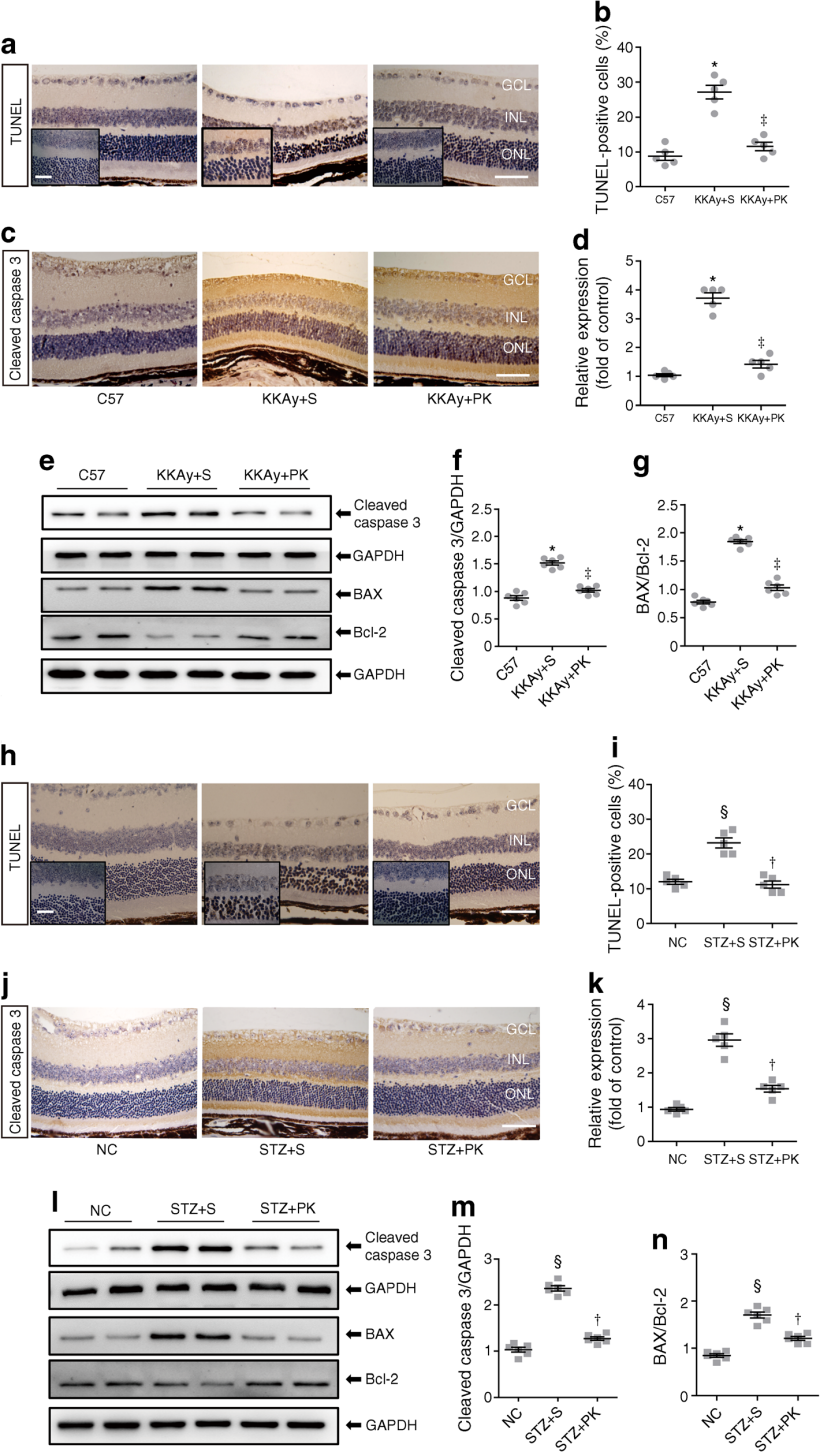
Pancreatic kallikrein improves retinal apoptosis in KKAy and HFD/STZ-induced type 2 diabetic mice. (**a**) Representative images of TUNEL staining in the C57, KKAy + saline (S) and KKAy + pancreatic kallikrein (PK) groups. (**b**) Quantification of the number of TUNEL-positive cells in six randomly selected fields. (**c**) Representative immunohistochemical micrographs of retinas stained for cleaved caspase 3 in the three groups and (**d**) relative expression of cleaved caspase 3. *n*=5 experimental samples per group. (**e**) Western blot analysis of cleaved caspase 3, BAX and Bcl-2 protein, with GAPDH run for each gel. (**f**, **g**) Densitometric analysis for band intensities normalised to GAPDH in each experimental group shown. *n*=6 experimental samples per group. (**h**, **i**) Representative photomicrographs of TUNEL staining and the percentage of TUNEL-positive cells in the normal control, STZ + S and STZ + PK groups. (**j**, **k**) Immunohistochemical micrographs of retinas stained for cleaved caspase 3 and the relative expression in the three groups. *n*=5 experimental samples per group. (**l**) Western blot analysis of cleaved caspase 3, BAX and Bcl-2, with GAPDH run for each gel, and (**m**, **n**) densitometric analysis for band intensities normalised to GAPDH in each experimental group shown. *n*=6 experimental samples per group. Scale bars, 50 μm in (**a**, **c**, **h**, **j**); inset boxes in (**a**, **h**) indicate enlarged images, scale bars, 20 μm. Data are expressed as means ± SEM. Fold of control data are calculated using the control for that experiment (C57 or NC). *p<0.05 vs the C57 group; ^‡^*p*<0.05 vs the KKAy + S group; ^§^*p*<0.05 vs the normal control (NC) group; ^†^*p*<0.05 vs the STZ + S group. GAPDH, glyceraldehyde 3-phosphate dehydrogenase; GCL, ganglion cell layer; INL, inner nuclear layer; NC, normal control; ONL, outer nuclear layer; PK, pancreatic kallikrein; S, saline

Many studies have shown that oxidative stress and inflammation can induce apoptosis in diabetic retinopathy [21, 22]. To verify whether pancreatic kallikrein can protect against diabetic retinopathy by inhibiting apoptosis, we first used a TUNEL assay to detect apoptosis. The proportion of apoptotic cells was significantly increased in the KKAy + saline group compared with the C57 group, while the addition of pancreatic kallikrein attenuated this increase (Fig. 6a, b). Next, we examined the expression of cleaved caspase 3 using immunochemical staining. We found that cleaved caspase 3 expression was significantly higher in KKAy + saline mice than in C57 mice, while it was notably decreased in KKAy + pancreatic kallikrein mice (Fig. 6c, d). Furthermore, western blot analysis also revealed that pancreatic kallikrein decreased the levels of cleaved caspase 3 and attenuated increases in the BAX/Bcl-2 ratio (Fig. 6e–g). These results suggest that pancreatic kallikrein may protect against diabetic retinopathy by inhibiting apoptosis. The results for HFD/STZ-induced diabetic mice were consistent with those for KKAy mice (Fig. 6h–n).

### Pancreatic kallikrein activates expression of the KKS

**Fig. 7:**
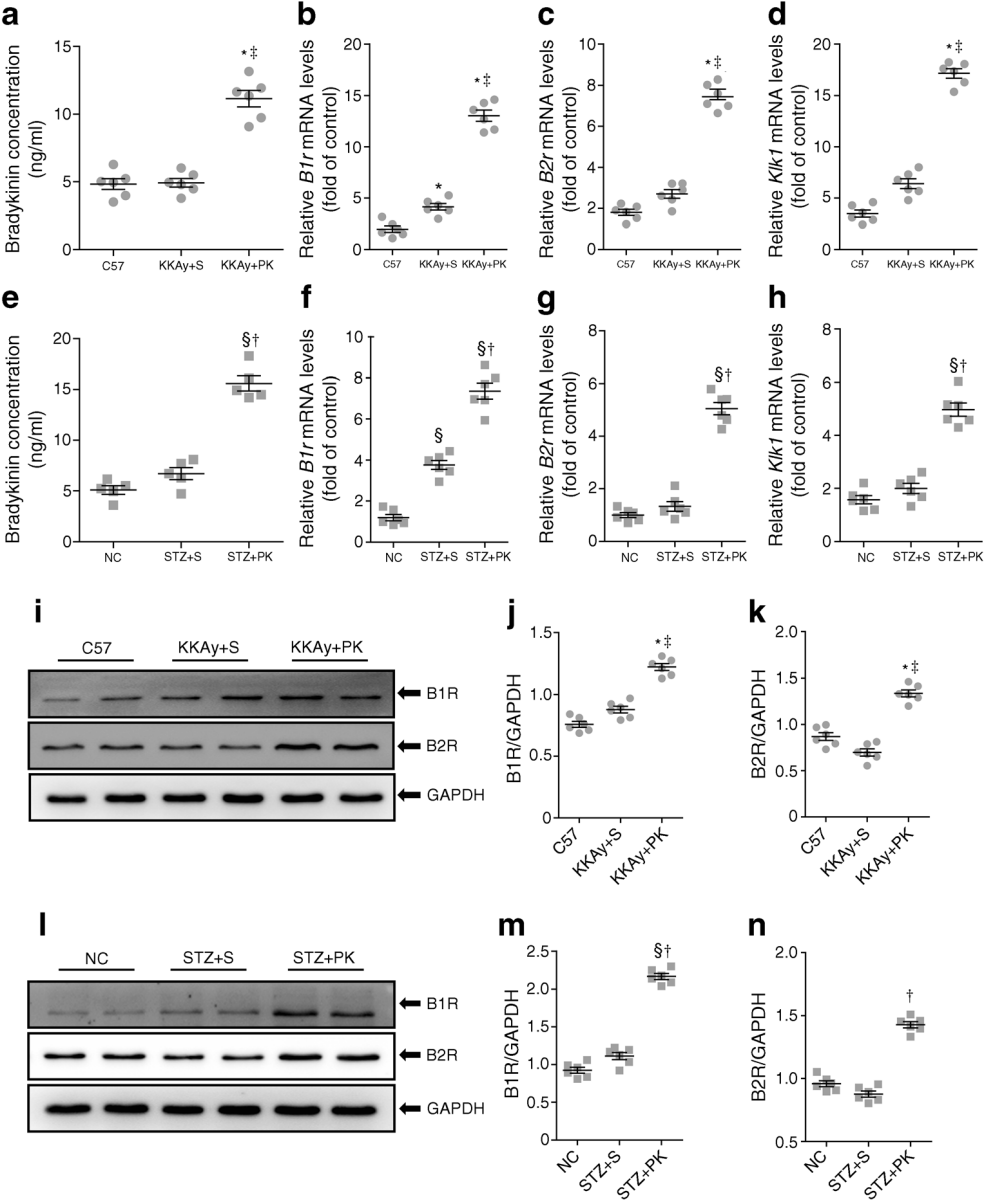
Pancreatic kallikrein increases the expression of the KKS. (**a**, **e**) Retinal bradykinin levels were measured by ELISA in each experimental group. qPCR analysis for the expression of *B1r*, *B2r* and *Klk1* in (**b**–**d**) KKAy and (**f**–**h**) HFD/STZ-induced type 2 diabetic mice, and their controls. (**i**, **l**) Western blot analysis of B1R and B2R and (**j**, **k**, **m**, **n**) densitometric analysis of band intensities normalised to GAPDH in each experimental group. Fold of control data are calculated using the control for that experiment (C57 or normal control [NC]). Data are expressed as means ± SEM; *n*=5−6 experimental samples per group. *p<0.05 vs the C57 group; ^‡^*p*<0.05 vs the KKAy + saline (S) group; ^§^*p*<0.05 vs the NC group; ^†^*p*<0.05 vs the STZ + S group. GAPDH, glyceraldehyde 3-phosphate dehydrogenase; NC, normal control; PK, pancreatic kallikrein; S, saline

To verify whether pancreatic kallikrein affects the expression of the KKS, we first determined serum bradykinin levels by ELISA in KKAy mice. The results showed that the levels of bradykinin were increased after pancreatic kallikrein treatment. However, there was no significant difference between the C57 and KKAy + saline groups (Fig. 7a). Next, we examined gene and protein expression levels of retinal KKS components. Only *B1r* was statistically significantly different between the C57 and KKAy + saline groups. Pancreatic kallikrein significantly increased the mRNA expression of *Klk1*, *B1r*, and *B2r* (Fig. 7b–d). Consistent with the results of qPCR, the protein expression of B1R and B2R was significantly increased after pancreatic kallikrein. However, there was no significant difference between the C57 and KKAy + saline groups (*p* = 0.2; *p* = 0.27) (Fig. 7i–k). Similar results for the KKS in HFD/STZ-induced diabetic mice are shown in Fig. 7e–h, l–n. Therefore, the results suggest that the observed upregulation of B1R and B2R was due to exogenous supplementation with pancreatic kallikrein, resulting in elevated levels of bradykinin.

## Discussion

The KKS is a complex and multifunctional endogenous peptidergic system consisting mainly of kininogen, serum or tissue kallikreins, bradykinin and kinin. Among these constituents, kallikrein is a key enzyme of the KKS; it was originally found in human urine and used as a vasodilatory substance in antihypertensive therapy [23, 24]. There are two major classes of kallikrein, plasma kallikrein and tissue kallikrein, which differ greatly in their molecular mass, substrate, immunological properties, gene structure and type of kinins released. The effects of both classes on diabetic retinopathy are also very different. Some studies have suggested that plasma kallikrein contributes to retinal vascular dysfunctions in diabetic rats [13, 25] and that its inhibitors can improve diabetic macular oedema [26]. Other studies have shown that tissue kallikrein improves diabetic retinopathy by inhibiting retinal vascular permeability and VEGF increases in diabetic rats [14]. Therefore, our study mainly explored whether pancreatic kallikrein could protect against retinopathy and the possible mechanisms by which this might occur.

In this study, we selected two mouse models of type 2 diabetes for further validation. One was the KKAy mouse model, which was developed by Japanese scholars who transferred the yellow obese *Ay* gene into KK mice. These mice exhibit hyperglycaemia, severe obesity, hyperlipidaemia and insulin resistance, and are considered to be spontaneous type 2 diabetic mice [15, 27]. Many studies have shown that KKAy mice develop proteinuria, mesangial matrix accumulation and glomerular basement membrane thickening, making them good models for studying diabetic nephropathy [28, 29]. Because diabetic nephropathy and retinopathy involve microvascular lesions, the pathological changes are similar. Therefore, KKAy mice have also been used to study diabetic retinopathy [30]. Our second model in this study used an HFD combined with STZ injection to induce type 2 diabetes. This model is the most widely accepted animal model for evaluating retinal complications in type 2 diabetes [31–33]. Our results showed that KKAy mice developed hyperglycaemia (blood glucose >16.7 mmol/l) after 12 weeks, and most mice in the HFD group developed hyperglycaemia (blood glucose >16.7 mmol/l) after i.p. injection of STZ. Therefore, both models were successfully established and were in the early stages of diabetic retinopathy due to the short study duration.

Our results demonstrated that pancreatic kallikrein was unable to improve metabolic abnormalities such as body weight, blood glucose, liver function, renal function or lipids in either diabetic mouse model. However, pancreatic kallikrein significantly improved retinal pathological structural features, increasing retinal thickness and ameliorating retinal acellular vessel formation and pericyte loss in both models. The results from the two models were similar, but there were some differences such as in body weight and ALT, which may have resulted from differences in genetic background, individual variations among different mice, sensitivity to STZ or severity of hyperglycaemia. After confirming the retinal protective effect of pancreatic kallikrein, we explored the underlying mechanism. Hyperglycaemia leads to increased production of ROS in the body. The retina is particularly sensitive to oxidative stress because of its high polyunsaturated fatty acid content, high consumption of oxygen and glucose oxidation [34]. Many studies have shown that oxidative stress is elevated in people with diabetic retinopathy and animal models of the disease, and that it plays a crucial role in the pathogenesis of diabetic retinopathy [5]. Liu et al demonstrated that kallikrein could inhibit nitrotyrosine and increase glomerular-stimulating hormone, inhibiting oxidative stress and thus improving diabetic nephropathy [11]. In agreement with the results of the above-mentioned studies, our results demonstrated that pancreatic kallikrein treatment could reduce the production of ROS, downregulating NOX2 and upregulating the antioxidant SOD2 in both type 2 diabetic mouse models.

In addition, many studies have shown that inflammation is a major pathogenic factor of diabetic retinopathy that can lead to retinal blood vessel loss and vascular leakage in the early stages of the disease [6]. Furthermore, hyperglycaemia-induced oxidative stress can also lead to the production of inflammatory factors such as TNF-α and IL-1β [35]. In this study, we found that vascular leakage occurred in both diabetic mouse models and that pancreatic kallikrein treatment attenuated the vascular permeability. Previous studies [36, 37] have shown that VEGF is an important proinflammatory factor that plays an important role in causing vascular leakage. Kato et al found that kallidinogenase (kallikrein) could inhibit VEGF expression and improve retinal vascular permeability [14]. Nakamura et al also found that tissue kallikrein inhibited retinal neovascularisation via the cleavage of VEGF_164_ in an oxygen-induced retinopathy model [38]. In agreement with the results of the above-mentioned studies, our results demonstrated that VEGF expression was elevated in the diabetic groups but reduced by pancreatic kallikrein. We also examined the expression of the inflammatory cytokines TNF-α and IL-1β, and found that pancreatic kallikrein decreased the expression of these cytokines in the retina under diabetic conditions.

In addition to directly leading to diabetic retinopathy, oxidative stress and inflammation can also induce apoptosis, which further aggravates diabetic retinopathy [21]. Further, TNF-α also plays an important role in the loss of diabetic microvascular cells [39]. Therefore, we next explored the effect of pancreatic kallikrein on retinal apoptosis. Many studies have shown that loss of pericytes is an early and important change in diabetic retinopathy [40, 41]. In this study, we observed loss of pericytes and an increase in acellular capillaries in the two diabetic mouse models, and found that pancreatic kallikrein treatment improved these phenomena. In addition, analysis of TUNEL and apoptosis-related indicators (cleaved caspase 3 and BAX/Bcl-2 ratios) further confirmed that pancreatic kallikrein could improve apoptosis in diabetic retinopathy.

What effect does pancreatic kallikrein have on the KKS? Since pancreatic kallikrein acts primarily on kininogen to produce bradykinin, which acts on its receptors (B1R and B2R), we examined the expression of B1R and B2R. B1R is known to be minimally expressed under physiological conditions but strongly induced in pathological states; in contrast, B2R is constitutively expressed. Previous studies have shown that B1R and B2R expression is upregulated in high glucose-stimulated endothelial cells [42], vascular smooth muscle cells [43] and STZ-induced diabetic rats [44]. However, in our two diabetic models, the protein levels of B1R and B2R were not significantly different in the diabetic groups compared with the normal groups. This discrepancy may be due to differences in the animal models, the severity of hyperglycaemia or the duration of diabetes. Moreover, this study showed that the expression of B1R and B2R was significantly increased after pancreatic kallikrein treatment, indicating that the upregulation might be mediated by ligand-induced effects. Interestingly, some studies have shown that B1R is involved in the inflammatory cascade and in vascular permeability in diabetic retinopathy, and that inhibiting B1R can improve diabetic retinopathy [45–47]. The discrepancy between the results of those studies and ours may be due to differences in the animal models, genetic backgrounds or severity of hyperglycaemia. More importantly, the KKS and renin–angiotensin system are key proteolytic systems that control a wide spectrum of systemic and local physiological activities. Multiple interactions between these two systems indicate that they are co-dependent; changes in one system are unavoidable in the other. Kallikrein generates kinins and also converts prorenin to renin. Thus, it participates in both kinin-dependent and angiotensin-dependent pathways [48]. In addition, after systemic pancreatic kallikrein treatment, pancreatic kallikrein may act on kininogen to produce bradykinin, which is not only related to B1R but also closely related to the action of B2R. Therefore, this cascade effect is far different from the effect of direct application of B1R inhibitors or agonists. Moreover, Kakoki et al demonstrated that a lack of both bradykinin B1R and B2R can enhance nephropathy, neuropathy and bone mineral loss in Akita diabetic mice [49]. Further studies are warranted to examine the mechanism of the retinal protective role of pancreatic kallikrein in diabetic retinopathy.

In conclusion, our results demonstrate that pancreatic kallikrein has retinal protective effects in KKAy and HFD/STZ-induced type 2 diabetic mice. These effects include improving pathological structural changes in the retina, ameliorating diabetes-induced retinal oxidative stress and inflammation, and attenuating apoptosis. These effects may be attributed, at least in part, to upregulation of bradykinin receptors, but other mechanisms cannot be ruled out. Therefore, exogenous pancreatic kallikrein may represent a novel therapeutic agent for the early stages of diabetic retinopathy.

## Electronic supplementary material

ESM (PDF 455 kb)

## Data Availability

All data generated or analysed during this study are included in this article.

## Abbreviations

ALT: Alanine aminotransferase
B1R: B1 receptor
B2R: B2 receptor
HFD: High-fat diet
KKS: Kallikrein–kinin system
NOX2: NADPH oxidase 2
OCT: Optical coherence tomography
qPCR: Quantitative real-time PCR
ROS: Reactive oxygen species
SOD2: Superoxide dismutase 2
STZ: Streptozotocin
VEGF: Vascular endothelial growth factor
