# Supplementary material for: Pancreatic kallikrein protects against diabetic retinopathy in KK Cg-Ay/J and high-fat diet/streptozotocin-induced mouse models of type 2 diabetes
Source: Diabetologia. 2019 Mar 5;62(6):1074–86. doi: 10.1007/s00125-019-4838-9 (PMC6509079; doi:10.1007/s00125-019-4838-9)
Supplement: Supplementary file 1 — (PDF 455 kb) [file 125_2019_4838_MOESM1_ESM.pdf]

Electronic Supplementary Material [ESM]

Pancreatic kallikrein protects against diabetic retinopathy in KKCg-*A^y*/J and high-fat diet/streptozotocin-induced mouse models of type 2 diabetes

Ying Cheng[1] · Xiaochen Yu[1] · Jie Zhang[2] · Yunpeng Chang[1] · Mei Xue[1] · Xiaoyu Li[1] · Yunhong Lu[1] · Ting Li [1] · Ziyu Meng[1] · Long Su[2] · Bei Sun[1]* · Liming Chen[1]*

1. NHC Key Laboratory of Hormones and Development, Tianjin Key Laboratory of Metabolic Diseases, Metabolic Diseases Hospital & Institute of Endocrinology, Tianjin Medical University, 300070 Tianjin, China
2. The Second Hospital of Tianjin Medical University, Tianjin, China

Corresponding authors: Liming Chen and Bei Sun

NHC Key Laboratory of Hormones and Development, Tianjin Key Laboratory of Metabolic Diseases, Metabolic Diseases Hospital & Institute of Endocrinology, Tianjin Medical University, 300070 Tianjin, China

Email xfx22081@vip.163.com and sun_peipei220@hotmail.com

METHODS

KK Cg-*A*^{y}/J mice Male KK Cg-*A*^{y}/J (KKAy) mice (weight 25-29 g, 8 weeks old) were obtained from HFK Bio-Technology Co., Ltd. (Beijing, China). This strain was obtained by Japanese scholars who transferred the Ay gene into KK mice [15]. After they had acclimated for 2 weeks, the mice were randomly divided into two groups. One group underwent daily i.p. injections of saline (154 mmol/l NaCl;n=16,10 ml/kg, KKAy + saline group),while the other group underwent daily i. p. injections of pancreatic kallikrein (Qianhong Biochemical Pharmaceutical Company, Changzhou, China) (*n*=16, 40 U/kg, KKAy + pancreatic kallikrein group) for 12 weeks. Both groups of mice were fed KK feed（HFK Bio-Technology Co., Ltd，Beijing, China）, which consisted of 17.5% protein, 48.5% carbohydrate and 17.9% fat. In addition, age-matched C57BL/6J mice (n= 16, C57 group) were used as the control group.

HFD/STZ-induced type 2 diabetic mice For another type 2 diabetic mouse model, male C57BL/6J mice (weight 18-25 g, 8 weeks old) were obtained from HFK Bio-Technology Co., Ltd., and diabetes was induced as described previously [16]. First, sixteen mice were randomly chosen to receive a standard diet (n=16, NC group), while the others were fed a high-fat diet (n=35, HFD group) for 12 weeks. Standard diet and high-fat diet purchased from HFK Bio-Technology Co., Ltd. (Beijing, China). The HFD consisted of 78.7% standard diet, 10% glucose, 10% animal fat, 1% total cholesterol and 0.3% sodium cholate. Subsequently, mice in the HFD group were injected with STZ (30 mg/kg, Sigma-Aldrich, St.Louis, MO, USA) intraperitoneally for 7 consecutive days, while the mice in the NC group were injected intraperitoneally with citrate-phosphate buffer and served as the normal control group (n= 12, NC group). One week after injection, blood glucose levels were tested, and mice with random blood glucose levels above 16.7 mmol/l were considered type 2 diabetic mice. The diabetic mice were then randomly separated into two groups, a PK-treated group (n=16, STZ+PK group) and a diabetic group treated with saline (n=16, STZ+NS group). The injection method, dose and time of PK administration were the same as those for the KKAy mice. Three mice were excluded because their blood glucose did not reach the standard. After 12 weeks of PK treatment, all mice were sacrificed. Body weight and blood glucose were measured weekly until the end of this study.

The mice were housed two per cage in polycarbonate cages with corncob bedding at 20

± 4°C with a 12 h light/12 h dark cycle and 10% humidity. All experiments in this study were randomized. Researchers and animal caretakers were blinded for each group. All animal studies were conducted in accordance with the Guide for the Care and Use of Laboratory Animals of the National Institutes of Health as well as the Animal Welfare Act guidelines. The protocols were approved by the Ethical Committee of Tianjin Medical University.

Intraperitoneal glucose tolerance test

In the HFD/STZ-induced diabetic model mice, an intraperitoneal glucose tolerance test (IPGTT) was performed after 12 weeks of a high-fat diet to assess insulin resistance. Mice in both groups were fasted for 12h and then injected intraperitoneally with 10% (wt/vol.) glucose solution at 2 g/kg body weight. Blood samples were taken from the tail and measured with a glucometer (Roche, Basel, Germany) at 0, 15, 30, 60, 90 and 120 min after glucose injection. Then, a curve was drawn, and the area under the curve (AUC) was calculated.

Immunohistochemistry

After deparaffinization, antigen retrieval was performed with heated Tris-EDTA, and endogenous peroxidase was blocked with 3% (vol./vol.) hydrogen peroxide. The sections were incubated with primary antibodies against cleaved caspase3 (1:200; no.A2156; Abclonal， MA, USA) at 4°C overnight. The sections were stained with a diaminobenzidine (DAB) kit after incubation with HRP-conjugated secondary antibody. The stained sections were observed and imaged under a light microscope, and the staining was quantified with Image-Pro Plus 6.0 analysis software.

Retinal trypsin digestion

Retinal trypsin digestion was performed as previously described [17]. Briefly, eyeballs were fixed in 4% paraformaldehyde for at least 48 h. The retinas were isolated and washed overnight with gentle shaking followed by 3% (wt/vol.) trypsin (no.0458; Amresco 1:250; PA, USA) digestion for 45 minutes at 37°C. Then, vasculature was gently separated and stained with periodic acid Schiff (PAS). Acellular vessels and pericytes were quantified from six random fields per retina under ordinary light microscopy according to a documented protocol [18].

Retinal vascular immunofluorescence staining

Next, we performed retinal immunofluorescence staining. Briefly, eyeballs were fixed in 4%

paraformaldehyde for 40 min at room temperature. Then, the retinas were dissected under a dissection microscope and transferred to 4% paraformaldehyde for incubation at 4℃ overnight. Next, the retinas were permeabilized with permeabilization buffer (PBS pH 6.8, 1% (wt/vol.) BSA, 0.5% (vol./vol.) Triton X-100) and stained with isolectin B4 (1:500, no.121413; Thermo Fisher scientific, MA, USA) at 4℃ overnight. Finally, the retinas were fixed and sealed with DAPI. The slides were observed under a laser scanning confocal microscope (LSM 710, Carl Zeiss AG, Oberkochen, Germany). In each group, eight fields were randomly selected and capillary density was analysed with Image J (NIH, MD, USA) software.

TUNEL Assay

Apoptosis detection was performed using a TUNEL apoptosis detection kit (Biotin-labelled POD method, universal, Nanjing, China) according to the manufacturer’s instructions. Briefly, paraffin sections were deparaffinized, permeabilized with proteinase K, blocked with 3% (vol./vol.) hydrogen peroxide, linked with TDT enzyme, and labelled with streptavidin-HRP. Then, the sections were stained with a DAB kit. Images were obtained under a light microscope, and staining was quantified with Image-Pro Plus 6.0 analysis software.

Western Blot Analysis

We used highly efficient RIPA lysis buffer plus PMSF to lyse retinal tissue. Proteins from the different experimental groups were separated by SDS-PAGE and transferred to PVDF membranes. The membranes were blocked with 5% (wt/vol.) non-fat milk, incubated with primary antibodies at 4°C overnight, and subsequently incubated with HRP-conjugated secondary anti-rabbit/mouse antibodies (1:5000, ZB-2301, ZSGB-Bio, Beijing, China) at room temperature for 2 h. The primary antibodies targeted NOX2 (1:2000; no.ab129068; Abcam, Cambridge, UK),SOD2 (1:1000; no.ab13533; Abcam, Cambridge, UK), IL-1β (1:500; no.12242; Cell Signaling Technology, MA, USA), TNF-α (1:500; no.BS1857; Bioworld, MO, USA),VEGF (1:1000; no. ab46154; Cambridge, UK), cleaved caspase 3 (1:1000; no.A2156; ABclonal, MA, USA), BAX (1:500; no.2772S; Cell Signaling Technology, MA, USA), Bcl-2 (1:1000; no.3498; Cell Signaling Technology, MA, USA), B1R (1:500; no. bs-8675R; Bioss, Beijing, China), and B2R (1:1000; no. ab134118; Abcam, Cambridge, UK).The protein bands were visualized with electrochemiluminescence (ECL) Blotting Detection Reagents

(WBKLS0500; Millipore, MA, USA). Densitometry analyses of the bands were performed using Image J software (NIH, MD, USA). GAPDH (1:5000; no.AP0063; Bioworld, MO, USA) was used as a loading control.

Quantitative real-time PCR (qPCR)

Total RNA was extracted from retinal samples using TRIzol reagent (9108, TaKaRa, Biotech, Japan) according to the manufacturer’s instructions. Next, RNA was converted to cDNA by synthesis (AT301-03, TransGen Biotech, Beijing, China), and quantitative PCR was conducted on a CFX96 Real-Time PCR System (Bio-Rad, USA) with aSYBR Green PCR Reagent kit (AQ131-04, TransGen Biotech, Beijing, China). GAPDH was used as a housekeeping gene. All primers are shown in ESM Table 1.

ESM Table 1 Sequences of primers used for RT-PCR

	Forward(5’-3’)	Reverse(5’-3’)
B1r	CCATCAGTCAGGACCGCTAC	CAGGGACGACTTTGACGGAA
B2r	GAACCGGCTCGCTTGAGAAA	GGTCCCGTTAAGAGCAGACC
klk 1	TGAGCTCCAGTGTGTGAACC	CGCACAAGTGTCTTTGCCTC
Gapdh	GCTGAGTATGTCGTGGAGT	GTTCACACCCATCACAAAC

B1r, B1 receptor; *B2r*, B2 receptor; *klk1*, kallikrein 1; RT-PCR, Real-time quantitative polymerase chain reaction.

ESM Table 2 The power and *p* value for each experiment in this study

		KKAy mice		HFD/STZ induced T2DM	
		p value	power	*p* value	power
OCT Retinal thickness(μm)		0.0001	0.82015	-	-
HE Retinal thickness(μm)		< 0.0001	0.81712	< 0.0001	0.89404
Acellular capillaries		< 0.0001	0.87621	0.0011	0.81330
Pericytes		0.0060	0.80788	0.0015	0.83661
Peripheral region of retina		< 0.0001	0.87811	0.1748	0.36291
Central region of retina		0.2173	0.35114	0.2110	0.29010
ROS fluorescence		< 0.0001	0.81214	< 0.0001	0.82081
WB	N0X2/GAPDH	< 0.0001	0.91729	< 0.0001	0.92170
	SOD2/GAPDH	< 0.0001	0.89484	< 0.0001	0.88385
	TNF-α /GAPDH	< 0.0001	0.92038	< 0.0001	0.91182
	IL-1β /GAPDH	< 0.0001	0.91208	< 0.0001	0.91714
	VEGF/GAPDH	< 0.0001	0.92196	< 0.0001	0.92857
TUNEL		< 0.0001	0.82177	< 0.0001	0.81257
IHC(cleaved caspase3)		< 0.0001	0.85197	< 0.0001	0.84062
WB	Caspase3/GAPDH	< 0.0001	0.91496	< 0.0001	0.92882
	BAX/Bcl-2	< 0.0001	0.92936	< 0.0001	0.91430
ELISA bradykinin		< 0.0001	0.82708	< 0.0001	0.84605
PCR	B1R	< 0.0001	0.92962	< 0.0001	0.92442
	B2R	< 0.0001	0.92940	< 0.0001	0.92666
	KLK	< 0.0001	0.93161	< 0.0001	0.91615
WB	B1R/GAPDH	< 0.0001	0.91606	< 0.0001	0.93113
	B2R/GAPDH	< 0.0001	0.91252	< 0.0001	0.92663

ESM Table 3 Basic characteristics of KKAy mice

	C57(n=16)	KKAy+NS(n=16)	KKAy+PK(n=16)	F value	P value
BW (g)	25±1	39.71±2.22[a]	39.5 ±4.65[a]	20.42	< 0.0001
BG (mmol/l)	6.917±1.66	30.37±3.88[a]	30.93±2.96[a]	125.1	< 0.0001
ALT(mmol/l)	38.2±12.62	71.03±9.25[a]	65.95±4.03[a]	8.94	<0.05
Cr(umol/l)	11.74±2.06	20.7±2.52[a]	17.87±1.69[a]	18.90	< 0.005
TG (mmol/l)	0.994±0.37	2.64±0.94[a]	2.66±1.15[a]	6.10	<0.05

BW, body weight; BG, blood glucose ; ALT, Alanine aminotransferase; Cr, Creatinine; TG, Triacylglycerol; NS, saline; PK, pancreatic kallikrein

Data are mean ± SEM. [a] $p < 0.05$ versus C57 group.

ESM Table 4 Basic characteristics of HFD/STZ-induced diabetic mice

	NC(n=16)	STZ+NS(n=16)	STZ+PK(n=16)	F value	P value
BW (g)	23.91±2.34	25.18±2.89	24±3.32	0.67	0.5220
BG (mmol/l)	7.3±1.18	19.96±3.39[a]	19.93±5.48[a]	17.69	<0.0005
ALT (mmol/l)	39.94±16.22	120.4±47.44[a]	80.7±21.17	7.26	<0.05
Cr(umol/l)	11.74±2.06	14.23±0.74	14.37±1.93	3.43	0.0839
TG (mmol/l)	0.68±0.28	1.526±0.64[a]	1.633±0.62[a]	6.65	<0.05

BW, body weight; BG, blood glucose; ALT, alanine aminotransferase; Cr, Creatinine; TG, Triacylglycerol; NS, saline; PK, pancreatic kallikrein

Data are mean ± SEM. [a] $p < 0.05$ versus NC group.

ESM Fig. 1 Pancreatic Kallikrein treatment ameliorates retinal inflammation in KKAy and HFD/STZ-induced type 2 diabetic mice. (a, b) Western blotting bands and quantification of CD68 in mouse retina. n= 6 experimental samples per group. **(c)** Representative immunohistochemical micrographs of retinas stained for CD68 in KKAy mice. Scale bar, 50 μm. **(d, e)** Western blotting bands and quantification of CD206 in mouse retina. n= 6 experimental samples per group. **(f)** Representative immunohistochemical micrographs of retinas stained for CD206 in KKAy mice. Scale bar, 50 μm.*P < 0.05 versus the C57 group; ‡P < 0.05 versus the KKAy+NS group; §P < 0.05 versus the NC group; †P < 0.05 versus the STZ+NS group. NC, normal control; NS, saline; PK, pancreatic kallikrein. GCL, ganglion cell layer; INL, inner nuclear layer; ONL, outer nuclear layer.

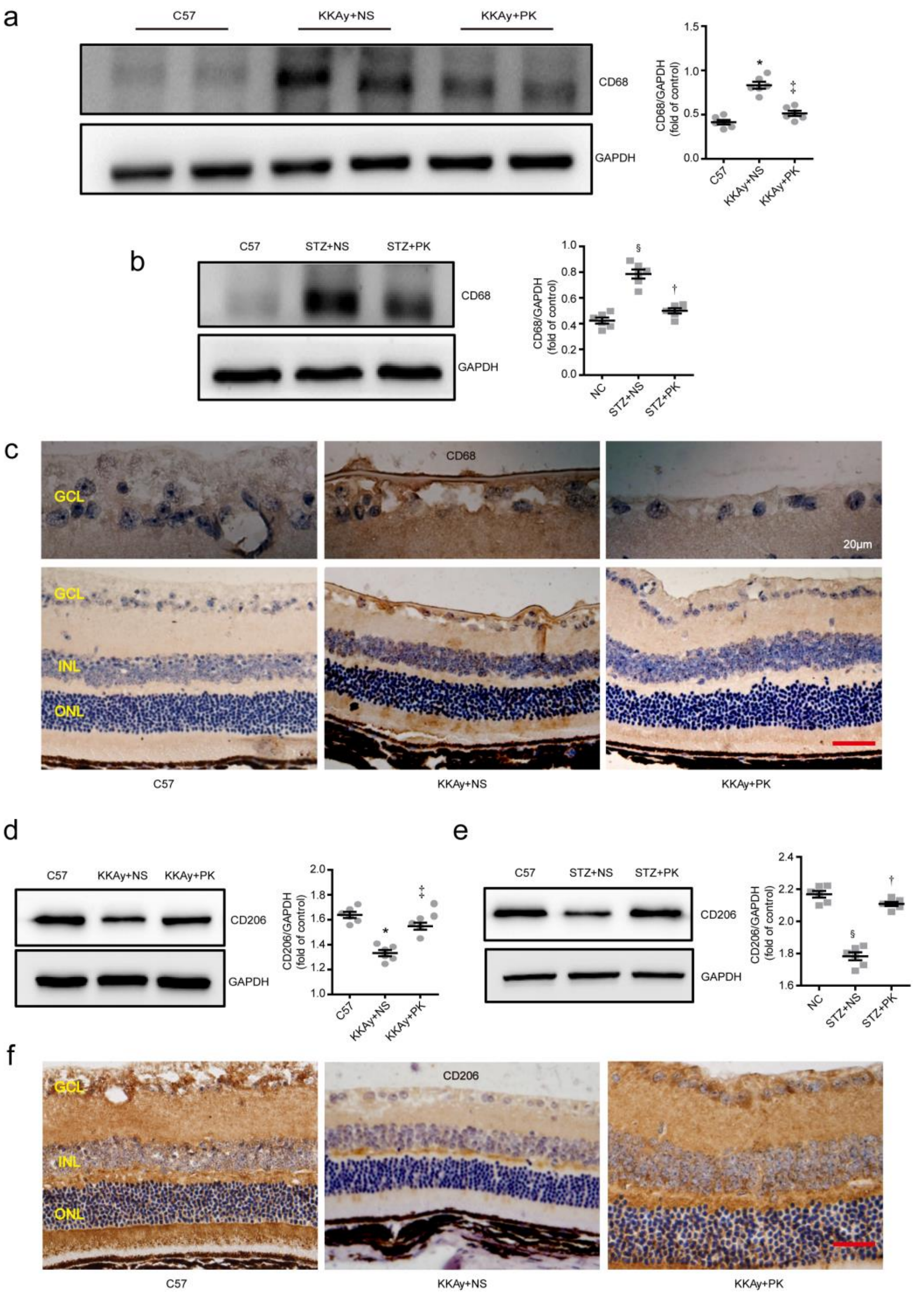

a
C57
KKAy+NS
KKAy+PK
CD68
GAPDH
CD68/GAPDH (fold of control)
b
C57
STZ+NS
STZ+PK
CD68
GAPDH
CD68/GAPDH (fold of control)
NC
c
CD68
GCL
INL
ONL
20μm
C57
KKAy+NS
KKAy+PK
d
C57
KKAy+NS
KKAy+PK
CD206
GAPDH
CD206/GAPDH (fold of control)
e
C57
STZ+NS
STZ+PK
CD206
GAPDH
CD206/GAPDH (fold of control)
NC
f
CD206
GCL
INL
ONL
C57
KKAy+NS
KKAy+PK
